# The consequences of porcine IVM medium supplementation with follicular fluid become reflected in embryo quality, yield and gene expression patterns

**DOI:** 10.1038/s41598-018-33550-4

**Published:** 2018-10-17

**Authors:** Piotr Pawlak, Ewelina Warzych, Adam Cieslak, Natalia Malyszka, Eliza Maciejewska, Zofia Eliza Madeja, Dorota Lechniak

**Affiliations:** 1 Department of Genetics and Animal Breeding, Poznan University of Life Sciences, Wolynska 33, 60-637 Poznan, Poland; 2 Department of Animal Nutrition, Poznan University of Life Sciences, Wolynska 33, 60-637 Poznan, Poland

## Abstract

Oocyte and embryo developmental competence are shaped by multiple extrinsic and intrinsic factors. One of the most extensive research areas in the last decade is the regulation of lipid metabolism in oocytes and embryos of different species. We hypothesized that differences in developmental competence of oocytes and embryos between prepubertal and cyclic gilts may arise due to distinct fatty acid profiles in follicular fluid. We found that supplementation of oocyte maturation media with follicular fluid from prepubertal pigs affected quality and development of embryos from prepubertal pigs while embryos of cyclic pigs were not affected. *PLIN2, SCD* and *ACACA* transcripts involved in lipid metabolism were upregulated in embryos originating from oocytes of prepubertal pigs matured with autologous follicular fluid. The surface occupied by lipid droplets tend to increase in oocytes matured with follicular fluid from prepubertal pigs regardless oocyte origin. The change into follicular fluid of cyclic pigs increased the efficiency of embryo culture and improved quality, while gene expression was similar to embryos obtained from cyclic gilts. We assume that the follicular fluids of prepubertal and cyclic pigs influenced the quality of oocytes and embryos obtained from prepubertal pigs which are more susceptible to suboptimal *in vitro* culture conditions.

## Introduction

Lipid metabolism is considered as one of the major factors responsible for shaping the developmental competence of oocytes and embryos. Porcine oocytes containing exceptionally high levels of intracellular lipids may serve as a model to study lipid metabolism during early embryonic development. Follicular fluid (FF), providing the natural environment for oocyte growth, serves as a supplemental source of fatty acids and growth factors to porcine oocyte *in vitro* maturation media (IVM)^1–3^. Qualitative and quantitative composition of FF may be directly translated into oocyte growth and maturation since it is influenced by animal age, nutrition and size of the follicle from which it was collected^2,3^. Moreover, the essential role of FF for oocyte nuclear maturation, cumulus cells expansion, apoptosis, polyspermy prevention and embryo culture efficiency has been documented^2,4,5^. A study of Grupen *et al*. showed the significance of FF origin for IVM of oocytes from prepubertal and adult gilts^6^. Earlier studies showed that the developmental competence of oocytes obtained from prepubertal gilts increase with the diameter of follicles from which they were collected^7–12^. This property however, has not been noted in oocytes collected from adult sows which show high competence despite the follicle origin.

The search for the causes of reduced developmental competence of oocytes obtained from prepubertal animals focused on different mammalian species, however without satisfactory results. A common observation was the reduced blastocyst yield, but the causes still remain mostly unknown. The efficiency of nuclear maturation *in vitro* did not provide the unequivocal answer, while the hypothesis on the insufficient cytoplasmic maturation is valid^13–15^. However, the influence of environmental factors on oocyte and preimplantation embryo quality have been well documented in other species^16–20^. Previous work on prepubertal (P) and cyclic (C) gilts has led to the discovery of differences in the amount of fatty acids (FA) in the follicular fluid^21^. Due to a well-known function of FAs and its influence on reproduction of farm animal species, we have decided to focus our current study on the domestic pig^22^. The majority of papers describing the composition of FF comes from studies done on cattle (impaired reproduction under the influence of negative energy balance) and humans (searching for markers of oocyte quality allowing to predict embryonic developmental potential). In both species, however, FF is not used for IVM therefore, the interpretation is limited to the effect of individual follicles on oocytes derived from them. The advantage of pig model, (10–20% v/v of FF in maturation medium), is that allows more experimental approach in elucidating how FF and its components influence full oocyte maturation and developmental competence. So far it has been shown that some FAs may negatively or positively affect the metabolism and the development of the embryo *in vitro*^23–27^. It is also known that the fate of the embryonic development does not solely rely on single molecules, but on their relative proportions, in a way that one FA can reduce or neutralize the inhibiting effects of another^3,26,28–33^. Therefore, our aim was to analyse the influence of FF collected from prepubertal and cyclic pigs (which differ in FA content) on lipid droplet (LD) number and on the area occupied by the droplets in relation to the expression of genes responsible for FAs metabolism in oocytes and embryos. Determining the role of FAs in FF will lead to better understanding of the lipid metabolism in ovarian follicle and potentially reveal new markers for prediction of oocyte developmental competence and embryo quality in IVF programs.

## Results

### *In vitro* embryo production (IVP)

The analysis of the effectiveness of embryo culture to the blastocyst stage showed an interesting correlation between applied FF and its effect on embryos. The least likelihood of embryos reaching the blastocysts stage was observed in experiments where oocytes collected from prepubertal (P) females were matured in IVM medium supplemented with the FF collected from the same experimental group (20.6%). However, if the same experimental group was supplemented with FF collected from cyclic (C) females a significant improvement in embryo culture efficiency was noticed (27.8%, P < 0.05; Table 1). Interestingly, no effect of FF was observed on cyclic embryos, and in both cases, the culture efficiency was high (C FF P — 29.6%, C FF C — 29.5%). It seems most likely that the FF collected from prepubertal animals showed no adverse effects on embryos, but FF from cyclic pigs improved conditions for oocyte maturation and improved the blastocyst yield.

**Table 1:** Total number of oocytes activated, the efficiency of blastocyst stage embryo production and quality of blastocysts as revealed by the cell number.

Group	Number Of Activated Oocytes	% Blastocyst/Oocytes	Mean Cell Number	Min/Max Cell Number
P FF P	315	20,6^a^	29,1^a^	18/51
C FF P	446	29,6^b^	44,6^b^	19/86
P FF C	312	27,8^b^	37,3^b^	25/50
C FF C	348	29,5^b^	37,0^b^	22/53
TOTAL	1421	26,9	37,4	18/86

Different letters indicate statistically significant differences between groups (P < 0.05).

The FF used for IVM influenced also the quality of blastocyst stage embryos revealed by the blastomere number. The lowest number of cells was observed in embryos obtained from prepubertal pigs after supplementation with the FF collected from the same group (29), but the change into FF of cyclic pigs resulted in a significant increase in the observed mean cell number (37, P < 0.05) comparable to embryos of cyclic gilts (37 cells – C FF C; 44 cells - C FF P). The FF collected either from P or from C pigs showed no adverse effects on embryos from cyclic females neither on the quality nor on the efficiency of embryo culture.

### Fatty acid profile and hormones concentration in follicular fluid

**Figure 1:**
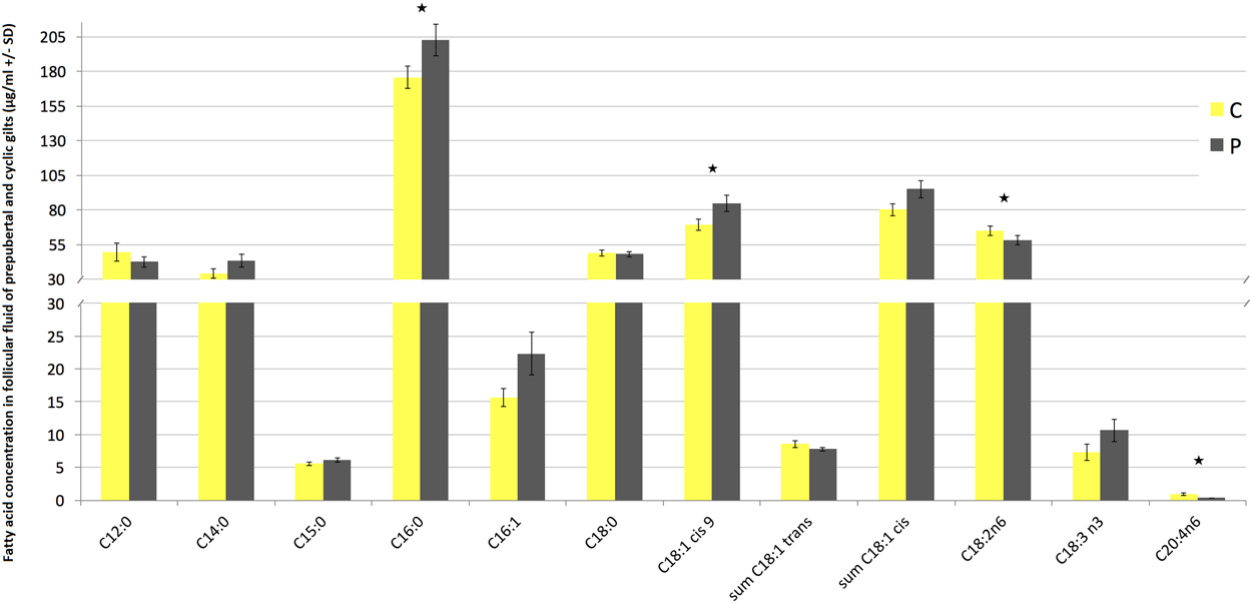
Fatty acid profile in follicular fluid of prepubertal (P) and cyclic (C) pigs (µg/ml). Asteriks indicate signifficant differences between groups (P < 0.05).

**Figure 2:**
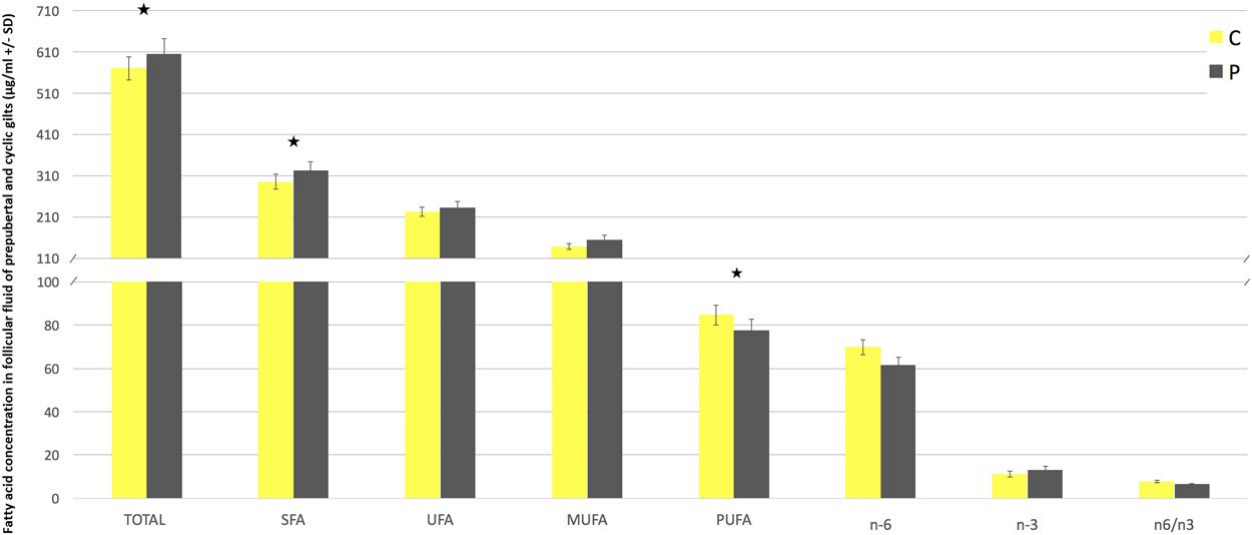
Fatty acid concentration (µg/ml) in follicular fluid of prepubertal (P) and cyclic (C) pigs classified into groups: SFA – saturated fatty acids; UFA – unsaturated fatty acids; PUFA – polyunsaturated fatty acids; MUFA – monounsaturated fatty acids; n6 – omega-6 fatty acids; n3 – omega-3 fatty acids. Asteriks indicate signifficant differences between groups (P < 0.05).

Fatty acid profile in P and C pigs differed in terms of total FA content, as well as palmitic, oleic, linoleic and arachidonic acids (Fig. 1). The highest total FA concentration was noted in FF of prepubertal pigs (P < 0.05) together with higher content of palmitic and oleic acids (P < 0.05). On the other hand, FF from cyclic pigs was characterized with increased content of PUFA (polyunsaturated fatty acids), linoleic and arachidonic fatty acids (P < 0.05, Fig. 2).

Progesteron and prostaglandin 2A (PGE2) concentration did not differ between FF collected from prepubertal and cyclic gilts (35.6+/−8.1 vs 39.5+/−9.6) and (19.2+/−14.4 vs 17.8+/−13.9) respectively. A significantly higher concentration of estradiol was found in FF of cyclic pigs (21.5+/−13.3 vs 9.1+/−5.9; P < 0.01). The differences in ratio of P4 to E2 was not significant between groups (3.9+/−1.33 vs 2.9+/−0.64).

### Lipid droplets

**Figure 3:**
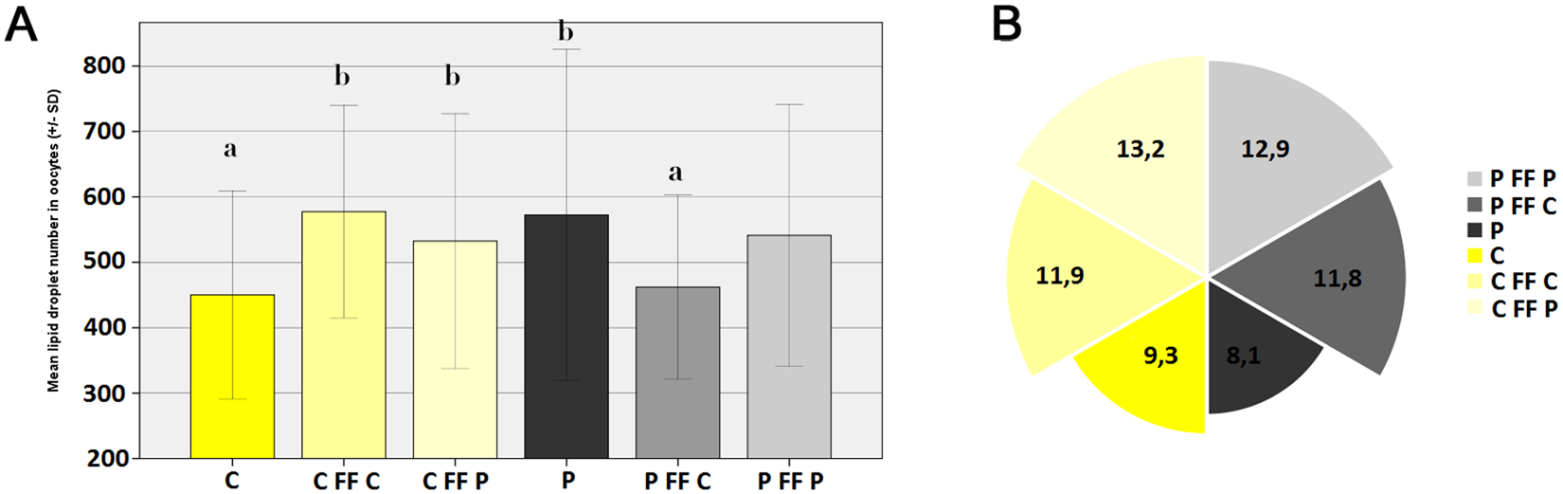
(**A**) The mean number of lipid droplets in all experimental groups as revealed by Bodipy 493/503 fluorescence staining; (**B**) the mean area occupied by lipid droplets in oocytes as revealed by ImageJ software measurements (μm^2^). Different letters indicate signifficant differences between groups (P < 0.05). P – oocytes from prepubertal gilts; (**C**) oocytes from cyclic gilts; P FF P – oocytes collected from prepubertal females matured with autologous, prepubertal follicular fluid; P FF C - oocytes collected from prepubertal females matured with follicular fluid from cyclic gilts; C FF P - oocytes collected from cyclic females matured with follicular fluid from prepubertal gilts; C FF C - oocytes collected from cyclic females matured with autologous, cyclic follicular fluid.

Statistical analysis (including a total of 268 oocytes, P - 123, C - 145) showed a significantly greater number of LD in oocytes of prepubertal gilts before IVM (P < 0.05). After *in vitro* maturation, the number of lipid droplets in oocytes of cyclic gilts increased significantly irrespective of the supplemented FF (P < 0.05). The number of lipid droplets in P oocytes did not change significantly after IVM if matured in P follicular fluid, however a significant decrease was noticed after the use of cyclic FF (Fig. 3A; P < 0.05). After IVM no significant differences has been noticed between experimental groups.

The results describing the area occupied by the LD showed lower variability than mean LD number. The oocyte area shared by lipid droplets was lowest before IVM and no correlation with gilt category was noticed (Table 2). After maturation, the area occupied by LD increased in all groups with noticeable trends (Fig. 3B). Maturation of oocytes in FF collected form prepubertal gilts resulted in increased but not statistically significant lipid droplet area in both P and C oocytes compared to cyclic FF (Table 2).

**Table 2:** Mean lipid droplet number and occupied area within the oocytes of each experimental group.

GROUP	Number Of Analysed Oocytes	Mean Lipid Droplet Number (Sd)	Min/Max	Area Occupied By Lipid Droplets (%)
C	48	450 (159)^a^	196/1088	9.3^a^
C FF C	40	577 (162)^b^	348/1104	11.9^a^
C FF P	35	533 (195)^b^	246/948	13.2^a^
P	59	572 (253)^b^	194/1178	8.1^a^
P FF C	39	462 (140)^a^	176/756	11.8^a^
P FF P	47	541 (200)^b^	224/1074	12.9^a^
TOTAL	268	522 (198)	176/1178	10.6
PRE-IVM	107	511 (223)	194/1178	8,6^a^
POST-IVM	161	528 (180)	176/1104	12.2^b^

Different letters indicate statistically significant differences between six experimental groups and between pooled PRE-IVM and POST-IVM groups (P < 0.05).

### Gene expression (mRNA level)

**Figure 4:**
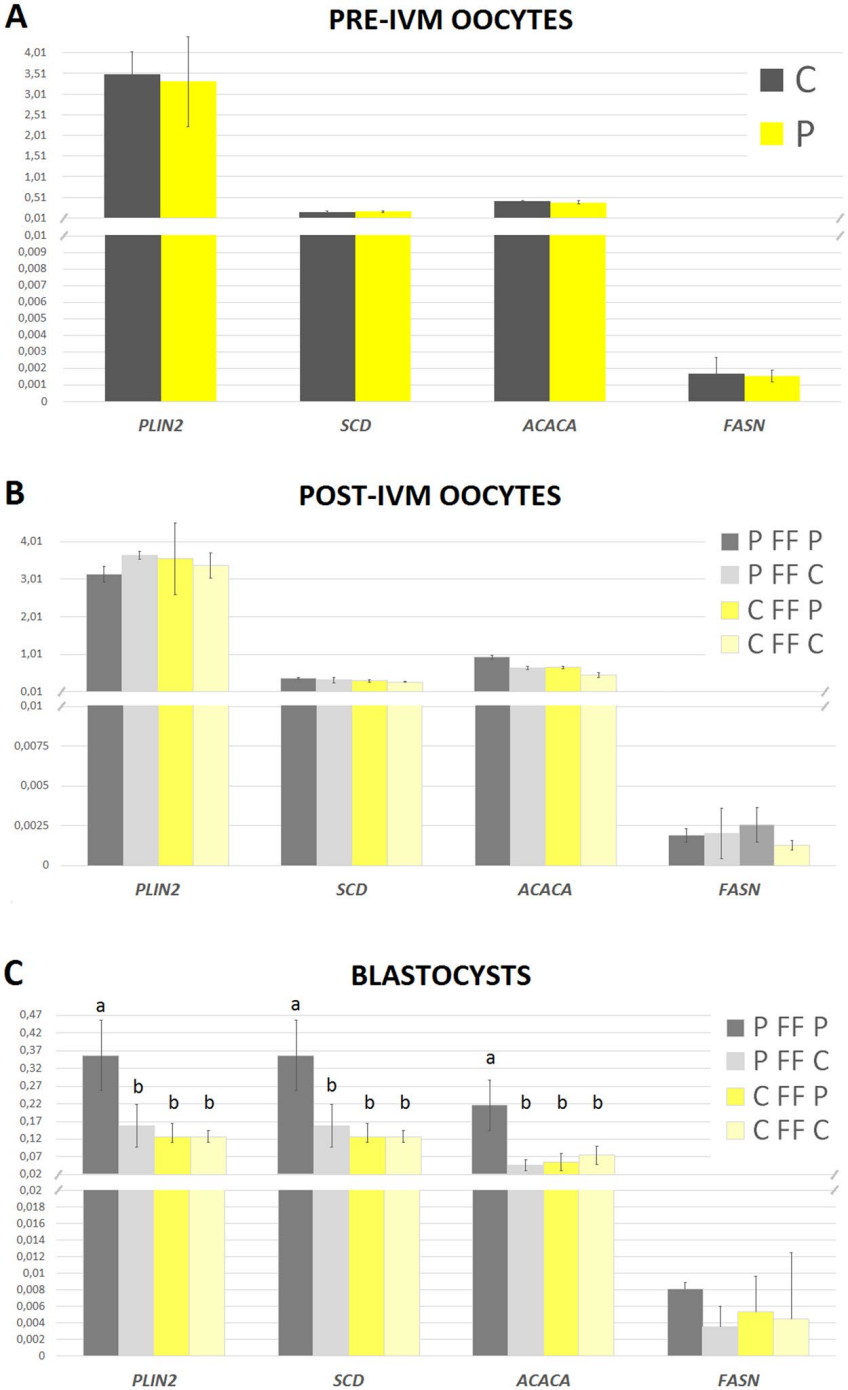
mRNA expression level of genes responsible for fatty acids metabolism in oocytes (pre-IVM), oocytes matured *in vitro* (post-IVM) and blastocyst stage embryos. Different letters indicate signifficant differences between groups within each analysed gene (P < 0.05). P – oocytes from prepubertal gilts; C – oocytes from cyclic gilts; P FF P – oocytes collected from prepubertal females matured with autologous, prepubertal follicular fluid; P FF C - oocytes collected from prepubertal females matured with follicular fluid from cyclic gilts; C FF P - oocytes collected from cyclic females matured with follicular fluid from prepubertal gilts; C FF C - oocytes collected from cyclic females matured with autologous, cyclic follicular fluid.

Gene expression on mRNA level was analysed for 26 oocyte samples (5 biological replicates before IVM in each group and 4 replicates after IVM). No significant differences in the expression level of genes related to developmental competence and quality (*BMP15, GDF9, OCT4*) were noted between groups before IVM and after IVM (Supplementary Fig. 3). Similarly, the analysis of genes related to fatty acid metabolism (*ACACA, SCD, FADS2, FADS1, FASN, PLIN2, ELOVL2, ELOVL6)* showed no statistically significant differences in mRNA expression level between oocytes of P and C pigs after IVM (P > 0.05, Fig. 4). No change in the gene expression was noticed independently of the fluid used for IVM.

The differences resulting from distinct IVM conditions were evident at the blastocyst stage of embryonic development. The *PLIN2, SCD*, *ACACA* gene expression was significantly increased (P < 0.05; Fig. 4) in blastocysts of prepubertal pigs originating from oocytes matured in FF of the same group compared to the other three groups. Supplementation of IVM with FF of cyclic pigs for maturation of oocytes from prepubertal pigs resulted in gene expression similar to embryos of cyclic pigs, which expressed above genes at comparable levels regardless the FF origin. Supplementation of IVM with FF from prepubertal pigs had no significant effect on *ELOVL2*, *ELOVL6*, *FASN, FADS1* and *FADS2* gene expression in embryos. The *OCT4* gene expression level did no differ between blastocysts of different groups (Supplementary Fig. 4).

### Immunofluorescent staining

**Figure 5:**
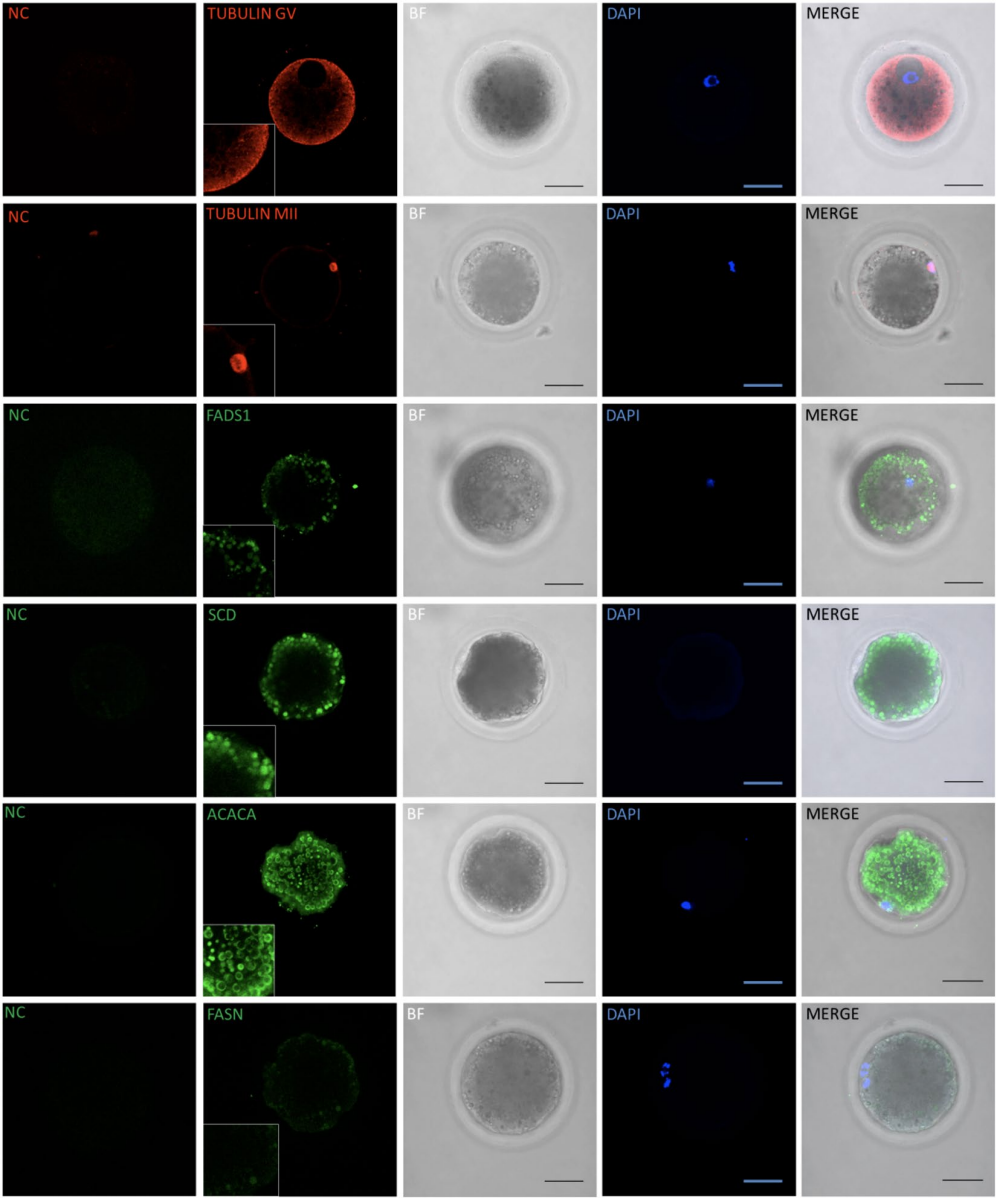
Immunofluorescence staining of oocytes for ACACA, FASN, FADS1, SCD and TUBULIN distribution patterns assessment. NC – negative control (without primary antibodies). BF – brightfield, DAPI – chromatin staining and merged channels. All stacks present (excluding marked GV tubulin staining) oocytes at MII stage of meiosis (visible and stained polar body). Scale bars represent 50 μm.

For the protein products of the several investigated genes we have observed distinct distribution patterns within oocytes and embryos. ACACA was found always on the surface of the lipid droplets while SCD, FADS1 colocalized with whole LDs with different intensity (Fig. 5). FASN foci were found closely localized next to some population of lipid droplets. No differences between oocytes before and after *in vitro* maturation have been noticed in distribution of analysed proteins. The same patterns like colocalization with lipid droplets or LD coating by these proteins were noticed in blastocyst stage embryos.

### Apoptosis

In all 47 examined embryos and 1944 blastomeres only few showed apoptotic signals. The frequency of apoptotic cells within parthenogenetic embryos was estimated on as few as 0.007% (Table 3). No correlation between oocyte origin and the frequency of apoptotic blastomeres was noticed.

**Table 3:** The incidence of apoptosis in embryos of different experimental groups.

Group	Number Of Analysed Embryos	Number Of Embryos With Apoptotic Nuclei	Number Of Apoptotic Cells	Total Cell Count
P FF P	11	4^a^	8^a^	436
C FF P	17	3^a^	4^a^	803
P FF C	9	0^a^	0^a^	298
C FF C	10	1^a^	1^a^	407
TOTAL	47	8	13	1944

Different letters indicate statistically significant differences between groups (P < 0.05).

## Discussion

The developmental potential of oocytes is shaped in a complex manner and controlled by multiple factors. In recent years, the metabolism of fatty acids and lipids in oocytes has been the subject of meticulous studies carried out in various mammalian species^34,35^. Our previous studies have indicated the possible link between the fatty acid content of the FF and the quality of oocytes from prepubertal and cyclic gilts. The pig is the only species in which the FF is added to the IVM media therefore, its impact is prolonged and the qualitative and quantitative FF content directly translates into the developmental potential of the oocytes.

Morphological evaluation of oocyte quality includes species-specific differences in cytoplasm granulation and coloration which reflect various lipid content^36^. However, the biological argumentation of lipid stores in mammalian oocytes remains no more than a hypothesis to explain litter size and preimplantation period in polytocous species^25,26,37^. Nevertheless, porcine oocytes are characterized by the highest lipid fraction among domestic species and thus extremely challenging for cryopreservation and intracytoplasmic sperm injection (ICSI)^38–42^. LDs form a functional unit with mitochondria, delivering fatty acids for the beta-oxidation and ATP synthesis essential for the further development^43^. Niu *et al*. showed that delipation of porcine oocytes affected the blastocyst yield however, it remains unknown whether this is related to a decrease in lipids stores, to mitochondria depletion or (as is most likely) to both phenomena^44,45^. Our study showed a significantly lower number of LDs in pre-IVM oocytes from cyclic pigs compared to prepubertal pigs, a status which may result from distinct growth environments and significantly lower total fatty acid content in FF from cyclic pigs. After IVM LD number increases in oocytes from cyclic pigs, irrespective of the FF used, however oocytes from prepubertal pigs decreased in LD number, reaching a level comparable to that of cyclic pigs but lower than the high LD number in these oocytes prior IVM. In cyclic pigs Milakovic *et al*. found a correlation between LD number in the oocyte and the diameter of the ovarian follicle^46^. This is interesting in the light of the earlier work of Bagg *et al*., which described no effect of follicle size on the developmental competence of the oocytes and embryos from cyclic pigs^7,9^. On the other hand, in prepubertal pigs, the developmental competence increases significantly with the diameter of ovarian follicles from which the oocytes were collected. Milakovic *et al*. also showed a significant decrease in LD count after IVM which may however arise due to the lack of FF supplementation, and in consequence to the depletion of stored lipids during extensive IVM^46^. Taken together, the differences in the number of LDs in cyclic oocytes do not correspond to the developmental potential and may not be a marker of their quality.

Unlike Milakovic *et al*., we noticed the great heterogeneity of LD number in all the groups of oocytes we examined^46^. Limiting the analysis to the LD number alone thus might not be very informative. Microscopic analysis also revealed a large variation in the LD size in oocytes. We therefore decided to look at this trait and the possible correlation between LD number and size, since they may change in size or shape, grow *de novo* or fuse and redistribute through the cell.^27,31,37,47^. We assumed that such analysis might more precisely describe the effect of particular FF or maturation conditions on oocyte characteristics. Our results clearly show that the LD area within post-IVM oocytes of C and P pigs significantly increased, indicating utilization of the FF components. We also observe higher LD areas in oocytes undergoing maturation with FF of prepubertal pigs, however, despite this marked trend, the difference was not significant. The percentage increase in LD area accounted for 1.1% and 1.3% in P and C oocytes, respectively. This indicates the important role of FF and its composition, which differs considerably depending on the follicles from which it is collected. It would thus be interesting to supplement IVM by FF with extreme amounts of FAs. Such approach would allow observation of the lipid accumulation, changes in LD area or the role of cumulus cells in preventing an the excess of FA supply, as has already been demonstrated in cattle^48^. Our results nevertheless show that the presence of FF in the maturation medium increased LD area which means that they are able to incorporate the lipids present in growth environment. This process is thought to be preferred by porcine oocytes and embryos for energy provision until placenta formation.

Considering the recent data of Grupen *et al*. it is interesting to ask which of the FF components provides optimal conditions for oocyte maturation, and whether such components act alone or synergistically. These authors found that oocytes from adult pigs were more readily recruited for capacitation than those from prepubertal pigs, possibly due to differences in FF steroid content^6^. Our results show that, although prepubertal FF does not seem to inhibit the competence of oocytes from cyclic pigs, it does not promote development of oocytes from prepubertal pigs which was clearly noted when FF from cyclic pigs was used for maturation. This leads to the conclusion that, once reached, full developmental competence cannot be revoked through the subtle physiological conditions provided by FF of prepubertal origin. However, it is possible to shape the quality of oocytes with reduced competence (e.g. prepubertal ones) using the growth environment supporting their maturation (e.g. cyclic FF). The FF of prepubertal pigs may thus be considered a quiet environment that supports the oocyte to the moment of achieving puberty by the donor and suppresses cytoplasmic maturation, unless matured in FF of cyclic pigs where the inhibiting effect is abolished. These data complement the research of Grupen *et al*., who presented similar observations by measuring only steroid content in the FF of prepubertal and adult gilts^6,9^. We did not see differences in progesterone and PGE2 concentration, but higher concentrations of estradiol in the FF of cyclic pigs was observed. Grupen *et al*. showed significant differences in progesterone and estradiol concentration in FF from prepubertal and adult pigs. The hormone contents observed by us and by Grupen are similar while the progesterone concentrations could be affected by the estrous cycle, during which it may change twenty-fold or by differences in animal age (adults vs. pubertal, cyclic). Interestingly, we noticed that the concentration of arachidonic acid, a product of PGE2 metabolism, was higher in cyclic pig FF, whereas prostaglandin concentration was not affected by FF origin. Regarding the results of Grupen *et al*. it should be noted that the pooling of FF might mask the action of some constituents (e.g., growth factors) that migh affect the cytoplasmic maturation.

Considering the standard requirement of the porcine oocytes to be matured with FF supplementation it is almost impossible to identify individual FAs, or a combination with a distinct, stimulating or inhibiting effect on the developmental competence of oocytes and embryos. This approach is routinely used in the bovine model and allows further interpretation of the physiological and pathological status of FF. It has been shown that palmitic and stearic acids negatively affect bovine oocytes and embryos^25^. Conversely, oleic and linoleic acids stimulate oocytes in development, but higher levels of these FAs may also inhibit the negative effects of saturated FAs^25,48^. Recently, the effect of various concentrations of nonesterified fatty acids (NEFAs) was investigated on *in vitro* maturation and *in vitro* embryo culture procedures in cattle, addressing the negative energy balance of postpartum cows under *in vitro* conditions^29,30,49^. The data on porcine FF are rather scarce. Our study pointed to significant differences in the concentration of particular FAs between prepubertal and cyclic FFs which may suggest that the environment around the time of oocyte *in vitro* maturation may have a programming impact on the developing preimplantation embryo^50^. To date numerous articles have addressed the effect of modified IVM conditions on embryo yield and physiology or cell count most often in cattle^16,18,51^. In our study, the embryos of prepubertal pigs were developing at lowest rate and exhibited the lowest number of cells when the autologous FF was used for IVM. However, the use of cyclic pig FF significantly increased the yield and quality of embryos to the levels of embryos from cyclic pigs, in which the quality and embryo culture efficiency was not affected by IVM conditions. Additionally, we have examined the occurrence of apoptosis in porcine parthenogenetic blastocysts, which is almost absent in comparison to IVF embryos or PA embryos of other species. The impact of maturation conditions has been found not only in physiological features of developing embryos, but also on the molecular level describing mostly expression of the genes responsible for cell division, developmental competence, transcription, apoptosis and methylation^20^. Only a few concerns genes related to lipid metabolism. Our data show that oocytes from prepubertal and cyclic pigs that underwent IVM with FF of various origins reached the MII stage at the same rate and expressed genes involved in lipid metabolism at similar levels. However, the differences arising from different maturation conditions were significantly manifested at the blastocyst stage. Interestingly, the selected genes controlling lipid metabolism were upregulated in embryos from the prepubertal pigs. Again, the use of FF from prepubertal pigs, with distinct FA profiles altered the expression pattern of the genes. Two genes (*ACACA*, the acetyl-CoA carboxylase and *FASN*, the fatty acid synthase) are involved in FA synthesis. The starting substrate for *ACACA* is acetyl-CoA which is transformed into malonyl CoA subsequently committing to the first step of FA synthesis^52^. The formation of new lipid droplets which stores lipids mostly in the form of triacyloglicerols is controlled by *ADRP* (also known as *PLIN2*). Palmitic acid, which is the most abundant in FF and oocytes, can be further converted into other FAs by a number of enzymes encoded by several genes (e.g. those encoded by *FADS1, FADS2, SCD, ELOVL2* and *ELOVL6*). The *PLIN2, ACACA* and *SCD* genes showed significantly higher expression in PA embryos of prepubertal pigs derived from oocytes matured in autologous FF. Replacing the FF in the same oocyte group by cyclic FF led to the downregulation of gene expression to the level observed in the embryos from cyclic pigs which expressed genes at the similar level regardless of IVM conditions. Interestingly, the genes related to maintenance of developmental competence and pluripotency were not differentially expressed between the evaluated groups of PA blastocysts. This suggests that higher FA concentrations, including saturated FAs in prepubertal FF, may have a regulatory effect on the expression of genes related solely to lipid metabolism during embryo development. Our results showed similar levels of *PLIN2* expression in oocytes of P and C pigs and no change following *in vitro* maturation, consistent with other results in porcine and bovine oocytes^53^. Perilipins were found in various tissues, while only *PLIN2* was present in oocytes of different species showing lipid droplet coating distribution. It may thus be the major functionally conserved component of LDs and thus serve as a marker of lipid metabolism in maturing oocytes. Little is known about the contribution of *PLIN2* to the development of the early mammalian embryo. However, increased expression of this gene in PA blastocysts of prepubertal pigs may reflect the growth environment during IVM and contribute to the formation of new LD and elevated lipid storage. The majority of data published on the effect of FA supplementation during IVM on gene expression comes from bovine research, where saturated fatty acid (SA) or high concentrations of NEFA (mixtures of palmitic, stearic and oleic acids) were used^25,54–57^. Interestingly, microarrays indicate changes in the lipid gene expression pathway, but also changes in cell metabolism involving changes in glucose consumption, resembling the mechanism of insulin resistance in somatic cells^28^. Van Hoeck *et al*. described increased expression of genes involved in lipid synthesis, FA uptake or cholesterol biosynthesis in bovine IVP blastocysts due to maturation media supplemented with high concentration of NEFA^24,28^. Our findings agree with the bovine data on the expression of *ACACA* which is upregulated in embryos of prepubertal pigs originating from oocytes matured with FF of higher FA content. Van Hoeck *et al*. found increased expression of *ACACA* and *ACSL1* in bovine blastocysts originating from oocytes matured with high NEFA concentrations^28^. *ACSL1* is involved in the activation of long chain fatty acids for B-oxidation, and also the synthesis of triglycerides. Parallel upregulation of these two genes thus suggests stimulated lipogenesis and higher lipid storage in LDs. Considering the metabolic pathway, one may further explain the increased expression of the *SCD* gene in embryos of prepubertal pigs. This gene plays an important role in regulating the expression of genes involved in lipogenesis, biosynthesis of membrane phospholipids and triglycerides and in regulating mitochondrial fatty acid oxidation^58^. The question however remains whether the observed alterations in gene expression are related to embryo development under suboptimal IVM conditions dissociable with oocyte competence or to a metabolic consequence during embryo growth.

In conclusion, the consequences of IVM medium supplementation with FF of distinct origin (prepubertal vs cyclic) became apparent at the blastocyst stage and concerned selected traits of embryo quality and blastocyst yield. Oocytes of prepubertal pigs matured in autologous FF developed to blastocyst at lower rate and displayed reduced cell counts and increased expression of *SCD, ACACA* and *PLIN2* genes (involved in fatty acid metabolism). Blastocyst yield and quality were improved by maturation of these oocytes in the FF collected from cyclic pigs. We assume that the distinct profiles of FAs in FF of prepubertal and cyclic gilts influenced the quality of oocytes of prepubertal pigs which are more susceptible to suboptimal *in vitro* culture conditions. With regard to oocytes of cyclic pigs, their quality was probably not affected by the FF origin due to full-bodied quality reflected by advanced cytoplasmic maturation. Moreover, the differences in FA content without modifying conditions such as diet or chemical intervention during IVM or IVC, are subtle, and do not seem to elicit the mechanism of lipotoxicity prevention. However, further study of the metabolome, secretome and defined media should reveal detailed influence of particular metabolites in the growth environment on mammalian oocytes and embryos.

## Material and Methods

### Collection of cumulus-oocyte complexes

The COCs used in experiment were collected post-mortem from porcine ovarian follicles of 3–6 mm diameter. Ovaries were transported in thermoisolated flask 2 hours after slaughter to the laboratory. Animals were 5–6 months old and weighted 100–110 kg. Ovaries were divided into two groups: P (prepubertal) and C (cyclic). P group was characterized by smaller size follicles and absence of corpus luteum, whereas C group COCs have more follicles >3 mm and presence of multiple corpus luteum. Immature COCs were aspirated from categorized ovaries with needle and syringe. COCs were placed in HEPES-Talp medium and scored morphologically. COC had evenly granulated cytoplasm and 4–5 layers of cumulus cells.

### Experimental groups

All procedures were performed in accordance with the “Act on the protection of animals used for scientific purpose” of the Republic of Poland, which complies with the European Union Legislation for the protection of animals used for scientific purposes. According to these regulations ethics approval was not required, as the biological material (ovaries) was collected upon animal slaughter in abattoir (Sokolow S.A. Robakowo).

COCs were divided in 6 groups. Two groups include oocytes before IVM: C – oocytes from cyclic pigs, P –oocytes from prepubertal pigs while four groups include oocytes after IVM with the supplementation of follicular fluid collected from both groups - C FF C (oocytes derived from cyclic gilts matured with cyclic follicular fluid), C FF P (oocytes derived from cyclic gilts matured with prepubertal follicular fluid), P FF C (oocytes derived from cyclic gilts matured with cyclic follicular fluid) and P FF P (oocytes derived from prepubertal gilts matured with prepubertal follicular fluid). The embryos at blastocyst stage were named the same as IVM oocytes.

### *In vitro* maturation

IVM media were supplemented with 10% v/v of the FF obtained either from cyclic or prepubertal pigs and of known fatty acids concentration. FF was aspirated from 3–5 mm ovarian follicles and collected to 1,5 ml tubes (Eppendorf, Germany) and centrifuged at 12000 rpm for 1 min. Supernatant was transferred to a new 1.5 ml tubes, frozen in liquid nitrogen and stored at −80 °C.

*In vitro* maturation of COC was performed in NCSU-23 (North Carolina State University Medium-23). Every COC group was incubated in 500 µl of IVM medium in four-well plates (Nunc, New York, USA) in HeraCell 150 incubator (Thermo Scientific) under conditions: 5% CO_2_ in atmosphere, 39 °C and maximum humidity. The first 20 h of IVM included IVM medium supplemented with hormones: 10U PMSG (pregnant mare serum gonadothropin, Chorulon, MSD Animal Health, Netherlands) and 10U hCG (Folligon, MSD Animal Health, Netherlands). Next step included transfer of COCs to fresh, equilibrated medium without hormones and incubation for 24 hours.

After IVM all oocytes with first polar body and no degenerative changes were denuded and transferred to PBS with 0.2% PVP (polyvinylpyrrolidone), frozen or fixed for further analyses. Oocytes subjected for embryo production were denuded in fresh equilibrated NCSU23 medium.

### Embryo production

*In vitro* matured oocytes (44 h) were denuded by pippeting and washed twice in fresh equilibrated NCSU23 medium (North Carolina State University Medium-23). Parthenogenetic activation (PA) was done by incubation of oocytes in TALP supplemented with 5 μM ionomycin for 5 min followed by incubation in 2 mM 6-DMAP (6**-**Dimethylaminopurine) for 4 h in 5% CO^2^, 5% O^2^ and 90% N^2^ (New Brunswick Galaxy 170 R, Eppendorf). Activated oocytes were transferred to 50 μl droplets of NCSU23 with 4 mg/mL BSA in groups of 20 and cultured to day 5 post activation (pa) when half of the medium was replaced with fresh NCSU23 supplemented with FBS (20% v/v). Embryos were cultured for 7 days and resulting blastocysts were fixed in 4% PFA for immunofluorescent staining and TUNEL analysis or frozen for gene expression analysis. Embryos subjected for IF or TUNEL served also for assessing the embryo quality by calculating number of cells.

### Lipid droplets staining

Oocysts were fixed in 4% PFA for 30 min at 37 °C in four-well plates (Nunc, New York, USA). PFA was removed by washing the oocytes twice in PBS with 0.2% PVP. Cells were stored at 4 °C no longer than two weeks. Oocytes were permeabilized in 0.2% Triton X-100 solution for 30 min. at RT and washed 2x times in 0.2% PVP/PBS. Fluorescent dye used to stain lipid droplets was 20 μg/ml BODIPY 493/503 (Life Technologies). Incubation was performed in 500 μl of dye solution in PBS at room temperature for one hour. The nucleus and the polar body were visualized by staining the oocytes with 0.5 μg/ml DAPI (4′,6-diamidino-2-phenylindole; Vector Laboratories, Burlingame, CA, USA). Stained and washed oocytes were mounted on glass slide with single concave (Comex, Poland), coverslipped and stored at 4 °C. Oocytes were analysed using confocal microscope Zeiss LSM 510 using 488 nm filter with band pass 500–550 nm for Bodipy 493/503 (Laser Argon2) and 420–480 nm for DAPI (Laser Diode 405). Lipid droplets were assessed through several optical sections of 3μm thickness each (Z-stack) captured every 10 μm to exclude double positioning of the same structures on two stacks (Supplementary Fig. 1). Every oocyte was captured from equatorial section to the top of the cell. Objective (Plan Neofluar 40x/1.3 Oil DIC; Zeiss, Germany), pinhole, filters, offset settings were kept constant throughout the experiments. Lipid droplets were counted using AxioVision software and “Events” tool followed by the creation of Excel file with final results for all oocytes. For the estimation of the area occupied by the LD in oocytes the ImageJ (NIH, USA) software was used. Firstly, the binary, 8-bit (black and white) photo were created. After setting the threshold for fluorescent signals the “Watershed” tool was used to separate the overlapping lipid droplets (Supplementary Fig. 5). The lipid droplet area (μM^2^) from all analysed stacks was dived by the oocyte area (from the corresponding stacks) giving final result in the percentage share of the lipid droplet occupied area within the oocyte.

### Fatty acid profile and hormone concentration in follicular fluid

Follicular fluid for FA composition analysis was collected by follicle aspiration from ovary pairs collected in abattoir. Each slaughtered gilt was subjected to removal of the reproductive tract, from which the ovaries have been excised and placed in a separate plastic container for transportation. FA profile and hormone concentration were measured in the same FF samples. Altogether 79 ovary pairs (48 prepubertal and 31 cyclic) were used for the experiment. Fatty acid composition of the FF was analyzed by gas chromatography according to the procedure described previously^21^. Estradiol (High Sensitivity; ADI-900–174), progesterone (ADI-900-011) and prostaglandin 2A (PGE2 High Sensitivity; ADI-930-001) concentration was measured using ELISA kits (Enzo Life Science) and protocol provided by the manufacturer. 50 μl of follicular fluid was used for analysis on Biotek Synergy2 plate reader. For each reaction seven standards have been prepared to build the standard curve, the blank and the NSB and TA wells with conjugate and antibody respectively. All samples were analysed in single assay in order to avoid variations.

### Apoptosis analysis

TUNEL was carried out on parthenogenetic day 7 blastocysts (7pa). The terminal TUNEL assay kit was used to detect apoptotic cells (DeadEnd^TM^ Fluorometric TUNEL system, Promega Biosciences Inc., Madison, WI, USA). Briefly, embryos were fixed in 4% paraformaldehyde solution in PBS for 30 min at 4 °C and washed twice in PBS + 0.025% PVP. Afterwards they were permeabilized by incubation in 0.2% Triton® X-100 solution in PBS for 5 min, washed twice in PBS + 0.025% PVP and covered with equilibration buffer provided by the kit. After 15 min, embryos were transferred to 50 µl equilibration buffer drop containing 1 µl of TdT (terminal deoxynucleotidyl transferase) and 5 µl of fluorescein-conjugated dUTP mix and covered with filtered paraffin oil. One-hour incubation at 37 °C in a dark chamber was terminated by 15 min washing in 2x SSC. The embryos were washed three times in PBS + 0.025% PVP and placed on slides in groups of 5–10. Slides were mounted with 40 µl of antifade medium containing DAPI (Vectasield, Vector Labs, Burlinghame, CA, USA), covered with glass coverslip and stored at 4 °C for maximum 2 weeks, before evaluated. Embryos pre-treated with DNase I (5 U/50 µl; Promega Biosciences Inc.) served as positive controls, whereas those not subjected to terminal TdT transferase, served as negative controls. Slides were evaluated with fluorescence microscope (Zeiss Axiovert 2.0) equipped with two filters for fluorescence detection: one to view the green fluorescence of fluorescein at 520 ± 20 nm and another for blue DAPI at 460 nm. A blastomere was considered as TUNEL positive when a strong, green fluorescence signal was observed (Supplementary Fig. 2).

### mRNA gene expression

Gene expression on mRNA level was performed on oocytes before and after IVM and embryos at blastocyst stage. Denuded oocytes (25 per sample) and embryos (3 per sample) were placed in 1.5 ml DNA LoBind tubes (Eppendorf) in PBS and frozen in liquid nitrogen. All samples were stored at −80 °C. The total RNA was extracted with mirVANA Paris Kit (Ambion, ThemoFisher Scientific) according to the manufacturer’s protocol. Briefly, the samples were incubated in Cell Disruption Buffer and 2x Denaturing Solution. Afterwards the acid phenol chloroform was added to cell lysate and centrifuged for 15 min at 14000 rpm. The upper, clear phase was mixed with isopropanol (1.25 v/v) and placed in a filter column and centrifuged at 8000 g for 1 min. The next steps involved 2x washing to remove contaminants. Finally, the total RNA was eluted into a fresh 1.5 ml LoBind tube with 100 μl of prewarmed Elution Buffer. The RNA samples were further precipitated with NF Pellet Paint Co-Precipitant. 1 μl of Pellet Paint, 10 μl of 3 M sodium acetate and 200 μl of freshly prepared 96% EtOH were added to RNA sample. After 5 min incubation, the samples were centrifuged at top speed for 10 min. The RNA pellet was washed followed by centrifugation by 75% and 96% EtOH and dried in 40 °C. RNA was resuspended in Molecular Biology Grade water (Sigma-Aldrich) in 10 μl. Next RNA was reverse transcribed using Transcriptor First Strand cDNA synthesis Kit (Roche) following manufactures protocol and using total isolated RNA. The protocol included denaturation of RNA and primers at 65 °C for 10 min followed by reverse transcription at 25 °C for 5 min and 42 °C for one hour and inactivation at 80 °C for 10 min. The cDNA samples were stored at −20 °C until further analysis. Genes responsible for fatty acid metabolism (*ACACA, ELOVL2, ELOVL6, SCD, FASN, FADS1, FADS2, PLIN2*) and developmental competence (*OCT4, GDF9, BMP15*) were analysed. Each cDNA sample was analysed in two independent PCR runs, and the mean value was used for the calculations of relative transcript abundance to the most stable reference genes *ACTB* and *GAPDH* (data not shown). List of analysed genes, primer and probe sequences designed by TibMolbiol (Germany) are shown in Supplementary Table 1. qPCR was conducted using the standard curve method. For this purpose, the desired sequences for all analysed genes were amplified by PCR and visualised on 1.5% agarose gel with the Gene Ruler^TM^ 100 bp DNA Ladder (Fermentas, Canada). The PCR product was excised from the gel, isolated and purified using the Gel Extraction Kit (Fermentas). Based on the DNA concentration measured with a Nanodrop c2000 system (Thermo Scientific, USA), a serial 10-fold dilutions of a DNA with a known concentration (standards) were generated. Each standard was used as a separate template for a real-time PCR reaction to produce the appropriate standard curve with the LightCycler 480 II software (Roche, Switzerland). The reaction conditions and efficiency of the reactions for all genes were analysed separately. All reactions were performed using the Light Cycler 480 II system with a set of supplied reagents. The 10 μl reaction mixture consisted of 5 μl of the LightCycler Probe Master, 0.5 μM primers, 0.3 μM probes and 1 μl each of the cDNA and. The reaction conditions were as follows: denaturation: 95 °C, 10 min; amplification: 40 cycles of 95 °C for 10 s, 60 °C for 10 s, 72 °C for 10 s; and final cooling at 40 °C. The temperature slope was set at 20 °C/s during amplification.

### Immunofluorescent staining

The oocytes and embryos for immunofluorescent staining were fixed in 4% PFA for 30 min at RT and stored in 4 °C. The applied staining protocol was previously described by Madeja *et al*. (Animal) Briefly the staining was initiated by permeabilization of oocytes and embryos for 20 min in 0.55% Triton X-100 in PBS followed by 10 min in NH_4_Cl and blocking for 60 min (10% FCS in PBS). Oocytes have been incubated with both primary (1:100) and secondary antibodies (1:200) at 4 °C for 24 h. Stained and washed oocytes or embryos were mounted on glass slide with single concave (Comex, Poland), coverslipped and stored at 4 °C. Slides were analysed using confocal microscope Zeiss LSM 880 using 488 nm filter with band pass 500–530 nm (Laser Argon2), 420–480 nm for DAPI (Laser Diode 405) and 543 nm with band pass 550 nm (HeNe1). Objective (Plan Neofluar 40x/1.3 Oil DIC; Zeiss, Germany), pinhole, filters, offset settings were kept constant throughout the experiments. Signal threshold (detector gain) for oocytes and embryos was set after examination of negative controls performed during every staining procedure. Negative control slides were prepared with exactly same protocol without incubation with primary antibody. A-Tubulin was used as a positive control indicating penetration of antibodies through zona pellucida and oolemma. ACACA, FASN, SCD and FADS1 localization was performed using Abcam and Santa Cruz antibodies (Supplementary Table 2).

### Statistical analysis

A comparison of the experimental groups was performed using IBM SPSS Statistics 23.0. All data (before computing) were subjected to testing for normal distribution using the Kolmogorov-Smirnov and Shapiro-Wilk tests. The differences in lipid droplet number and occupied area in oocytes of different groups were calculated using the Kruskal-Wallis and two-tailed Mann-Whitney U tests. mRNA gene expression differences were analyzed using nonparametric two-tailed Mann-Whitney U test. The frequency of apoptosis in oocytes of different groups were calculated using the chi-square and Fisher’s exact tests. Fatty acid and hormones concentration were analysed with t-Student and Mann-Whitney U tests respectively. All data with P < 0.05 were considered statistically significant.

## Electronic supplementary material

Supplementary Dataset 1

## References

[1] Sturmey RG, Reis A, Leese HJ, McEvoy TG (2009). Role of Fatty Acids in Energy Provision During Oocyte Maturation and Early Embryo Development. Reproduction in Domestic Animals.

[2] Bertoldo MJ (2013). Differences in the metabolomic signatures of porcine follicular fluid collected from environments associated with good and poor oocyte quality. Reproduction.

[3] Leroy J (2012). Intrafollicular conditions as a major link between maternal metabolism and oocyte quality: a focus on dairy cow fertility. Reproduction Fertility and Development.

[4] Ali A, Bousquet D, Sirard MA (2003). Origin of bovine follicular fluid and its effect during *in vitro* maturation on further development of bovine oocytes. Biology of Reproduction.

[5] Ducolomb Y (2013). Effect of porcine follicular fluid proteins and peptides on oocyte maturation and their subsequent effect on *in vitro* fertilization. Theriogenology.

[6] Grupen CG (2003). Relationship between donor animal age, follicular fluid steroid content and oocyte developmental competence in the pig. Reproduction Fertility and Development.

[7] Bagg MA, Nottle MB, Armstrong DT, Grupen CG (2007). Relationship between follicle size and oocyte developmental competence in prepubertal and adult pigs. Reproduction Fertility and Development.

[8] Bagg, M. A., Nottle, M. B., Armstrong, D. T. & Grupen, C. G. Differences in pre-pubertal and adult oocyte developmental competence is correlated with oocyte cAMP content in the pig. *Biology of Reproduction*, 236–236 (2005).

[9] Bagg MA (2004). Changes in ovarian, follicular, and oocyte morphology immediately after the onset of puberty are not accompanied by an increase in oocyte developmental competence in the pig. Theriogenology.

[10] Marchal R (2001). Meiotic and developmental competence of prepubertal and adult swine oocytes. Theriogenology.

[11] Marchal R, Vigneron C, Perreau C, Bali-Papp A, Mermillod P (2002). Effect of follicular size on meiotic and developmental competence of porcine oocytes. Theriogenology.

[12] Menino AR, Archibong AE, Li JR, Stormshak F, England DC (1989). Comparison Of *In Vitro* Development Of Embryos Collected From The Same Gilts At 1st And 3rd Estrus. Journal of Animal Science.

[13] Paczkowski Melissa, Bidwell Chris, Craig Bruce, Lipka Alex, Waddell Jolena, Krisher Rebecca (2007). EFFECT OF PUBERTY AND FOLLICLE SIZE ON GENE EXPRESSION IN PORCINE OOCYTES. Biology of Reproduction.

[14] Paczkowski M, Krisher R (2010). Aberrant Protein Expression Is Associated With Decreased Developmental Potential in Porcine Cumulus-Oocyte Complexes. Molecular Reproduction and Development.

[15] Paczkowski M, Terry D, Bidwell C, Krisher R (2006). Differential gene and protein expression in gilt and sow derived oocytes. Journal of Animal Science.

[16] Warzych E, Wrenzycki C, Peippo J, Lechniak D (2007). Maturation medium supplements affect transcript level of apoptosis and cell survival related genes in bovine blastocysts produced *in vitro*. Molecular Reproduction and Development.

[17] Kwon S (2015). Assessment of Difference in Gene Expression Profile Between Embryos of Different Derivations. Cellular Reprogramming.

[18] Rizos D, Ward F, Duffy P, Boland MP, Lonergan P (2002). Consequences of bovine oocyte maturation, fertilization or early embryo development *in vitro* versus *in vivo*: Implications for blastocyst yield and blastocyst quality. Molecular Reproduction and Development.

[19] Rizos D (2002). Analysis of differential messenger RNA expression between bovine blastocysts produced in different culture systems: Implications for blastocyst quality. Biology of Reproduction.

[20] Salilew-Wondim D, Tesfaye D, Hoelker M, Schellander K (2014). Embryo transcriptome response to environmental factors: Implication for its survival under suboptimal conditions. Animal Reproduction Science.

[21] Pawlak P (2012). No single way to explain cytoplasmic maturation of oocytes from prepubertal and cyclic gilts. Theriogenology.

[22] McKeegan PJ, Sturmey RG (2012). The role of fatty acids in oocyte and early embryo development. Reproduction Fertility and Development.

[23] Sirard MA (2012). Factors Affecting Oocyte and Embryo Transcriptomes. Reproduction in Domestic Animals.

[24] Van Hoeck V (2013). Oocyte developmental failure in response to elevated nonesterified fatty acid concentrations: mechanistic insights. Reproduction.

[25] Aardema H (2012). Oleic acid counteracts the negative effect of saturated fatty acids on bovine oocyte developmental competence. Reproduction in Domestic Animals.

[26] Prates E. G., Nunes J. T., Pereira R. M. (2014). A Role of Lipid Metabolism during Cumulus-Oocyte Complex Maturation: Impact of Lipid Modulators to Improve Embryo Production. Mediators of Inflammation.

[27] Prates EG (2013). Fatty acid composition of porcine cumulus oocyte complexes (COC) during maturation: effect of the lipid modulators trans-10, cis-12 conjugated linoleic acid (t10, c12 CLA) and forskolin. In Vitro Cellular & Developmental Biology-Animal.

[28] Van Hoeck V (2015). Interaction between differential gene expression profile and phenotype in bovine blastocysts originating from oocytes exposed to elevated non-esterified fatty acid concentrations. Reproduction Fertility and Development.

[29] Van Hoeck V (2012). The impact of elevated NEFA concentrations on mitochondrial function during bovine oocyte maturation. Reproduction in Domestic Animals.

[30] Van Hoeck Veerle, Sturmey Roger G., Bermejo-Alvarez Pablo, Rizos Dimitrios, Gutierrez-Adan Alfonso, Leese Henry J., Bols Peter E. J., Leroy Jo L. M. R. (2011). Elevated Non-Esterified Fatty Acid Concentrations during Bovine Oocyte Maturation Compromise Early Embryo Physiology. PLoS ONE.

[31] Fujimoto T, Ohsaki Y, Cheng J, Suzuki M, Shinohara Y (2008). Lipid droplets: a classic organelle with new outfits. Histochemistry and Cell Biology.

[32] Lonergan P (2003). Temporal sensitivity of bovine embryos to culture environment after fertilization and the implications for blastocyst quality. Reproduction.

[33] Lonergan P, Rizos D, Ward F, Boland MP (2001). Factors influencing oocyte and embryo quality in cattle. Reproduction Nutrition Development.

[34] Wonnacott KE (2010). Dietary omega-3 and-6 polyunsaturated fatty acids affect the composition and development of sheep granulosa cells, oocytes and embryos. Reproduction.

[35] Sinclair KD (2008). Amino acid and fatty acid composition of follicular fluid as predictors of *in-vitro* embryo development. Reproductive Biomedicine Online.

[36] Hunter MG (2000). Oocyte maturation and ovum quality in pigs. Reviews of Reproduction.

[37] Prates EG (2013). Fat area and lipid droplet morphology of porcine oocytes during *in vitro* maturation with trans-10, cis-12 conjugated linoleic acid and forskolin. Animal.

[38] Romek M, Gajda B, Krzysztofowicz E, Smorag Z (2008). A novel technique for evaluation of the lipid content in pig embryos. Reproduction Fertility and Development.

[39] Romek M, Gajda B, Krzysztofowicz E, Smorag Z (2007). Lipid composition of fat droplets of *in vivo*- and *in vitro*-produced porcine embryos. Reproduction Fertility and Development.

[40] Romek M, Gajda B, Krzysztofowicz E, Smorag Z (2006). Analysis of lipids in pig embryos produced *in vivo* and *in vitro*. Acta Biologica Cracoviensia Series Botanica.

[41] Romek M, Gajda B, Krzysztofowicz E, Smorag Z (2010). Changes of lipid composition in non-cultured and cultured porcine embryos. Theriogenology.

[42] McEvoy TG, Coull GD, Broadbent PJ, Hutchinson JSM, Speake BK (2000). Fatty acid composition of lipids in immature cattle, pig and sheep oocytes with intact zona pellucida. Journal of Reproduction and Fertility.

[43] Sturmey RG, O’Toole PJ, Leese HJ (2006). Fluorescence resonance energy transfer analysis of mitochondrial: lipid association in the porcine oocyte. Reproduction.

[44] Niu YJ (2015). Distribution and content of lipid droplets and mitochondria in pig parthenogenetically activated embryos after delipation. Theriogenology.

[45] Wang C (2015). Influence of Delipation on the Energy Metabolism in Pig Parthenogenetically Activated Embryos. Reproduction in Domestic Animals.

[46] Milakovic I (2015). Energy Status Characteristics of Porcine Oocytes During *In Vitro* Maturation is Influenced by Their Meiotic Competence. Reproduction in Domestic Animals.

[47] Thiele C, Spandl J (2008). Cell biology of lipid droplets. Current Opinion in Cell Biology.

[48] Aardema H., Lolicato F., van de Lest C. H. A., Brouwers J. F., Vaandrager A. B., van Tol H. T. A., Roelen B. A. J., Vos P. L. A. M., Helms J. B., Gadella B. M. (2013). Bovine Cumulus Cells Protect Maturing Oocytes from Increased Fatty Acid Levels by Massive Intracellular Lipid Storage. Biology of Reproduction.

[49] Pieterse MC (1991). Transvaginal Ultrasound Guided Follicular Aspiration Of Bovine Oocytes. Theriogenology.

[50] Sirard MA, Richard F, Blondin P, Robert C (2006). Contribution of the oocyte to embryo quality. Theriogenology.

[51] Warzych E, Peippo J, Szydlowski M, Lechniak D (2007). Supplements to *in vitro* maturation media affect the production of bovine blastocysts and their apoptotic index but not the proportions of matured and apoptotic oocytes. Animal Reproduction Science.

[52] Valsangkar DS, Downs SM (2015). Acetyl CoA Carboxylase Inactivation and Meiotic Maturation in Mouse Oocytes. Molecular Reproduction and Development.

[53] Zhang RN (2014). Expression of Perilipin 2 (PLIN2) in Porcine Oocytes During Maturation. Reproduction in Domestic Animals.

[54] Sutton-McDowall, M. L. *et al*. Nonesterified Fatty Acid-Induced Endoplasmic Reticulum Stress in Cattle Cumulus Oocyte Complexes Alters Cell Metabolism and Developmental Competence. *Biology of Reproduction* **94**, 10.1095/biolreprod.115.131862 (2016). 10.1095/biolreprod.115.131862 26658709

[55] Lolicato, F. *et al*. The Cumulus Cell Layer Protects the Bovine Maturing Oocyte Against Fatty Acid-Induced Lipotoxicity. *Biology of Reproduction* **92**, 10.1095/biolreprod.114.120634 (2015). 10.1095/biolreprod.114.120634 25297544

[56] van Hoeck V, Sturmey RG, Bols PEJ, Leroy J (2010). The effect of elevated non esterified fatty acid concentrations during bovine oocyte-complex maturation on subsequent embryo viability. Reproduction in Domestic Animals.

[57] Desmet, K. L. J. *et al*. Exposure of bovine oocytes and embryos to elevated non-esterified fatty acid concentrations: integration of epigenetic and transcriptomic signatures in resultant blastocysts. *Bmc Genomics* **17**, 10.1186/s12864-016-3366-y (2016). 10.1186/s12864-016-3366-y PMC5146907 27931182

[58] Moreau C, Froment P, Tosca L, Moreau V, Dupont J (2006). Expression and regulation of the SCD2 desaturase in the rat ovary. Biology of Reproduction.

