# Supplementary material for: The consequences of porcine IVM medium supplementation with follicular fluid become reflected in embryo quality, yield and gene expression patterns
Source: Sci Rep. 2018 Oct 17;8:15306. doi: 10.1038/s41598-018-33550-4 (PMC6193000; doi:10.1038/s41598-018-33550-4)
Supplement: Supplementary file 1 — Supplementary Dataset 1 [file 41598_2018_33550_MOESM1_ESM.docx]

**Supplementary DataSet**

**The consequences of porcine IVM medium supplementation with follicular fluid become reflected in embryo quality, yield and gene expression patterns.**

Piotr Pawlak ^1^*, Ewelina Warzych ^1^, Adam Cieslak ^2^, Natalia Malyszka ^1^, Eliza Maciejewska ^1^, Zofia Eliza Madeja ^1^, Dorota Lechniak ^1^


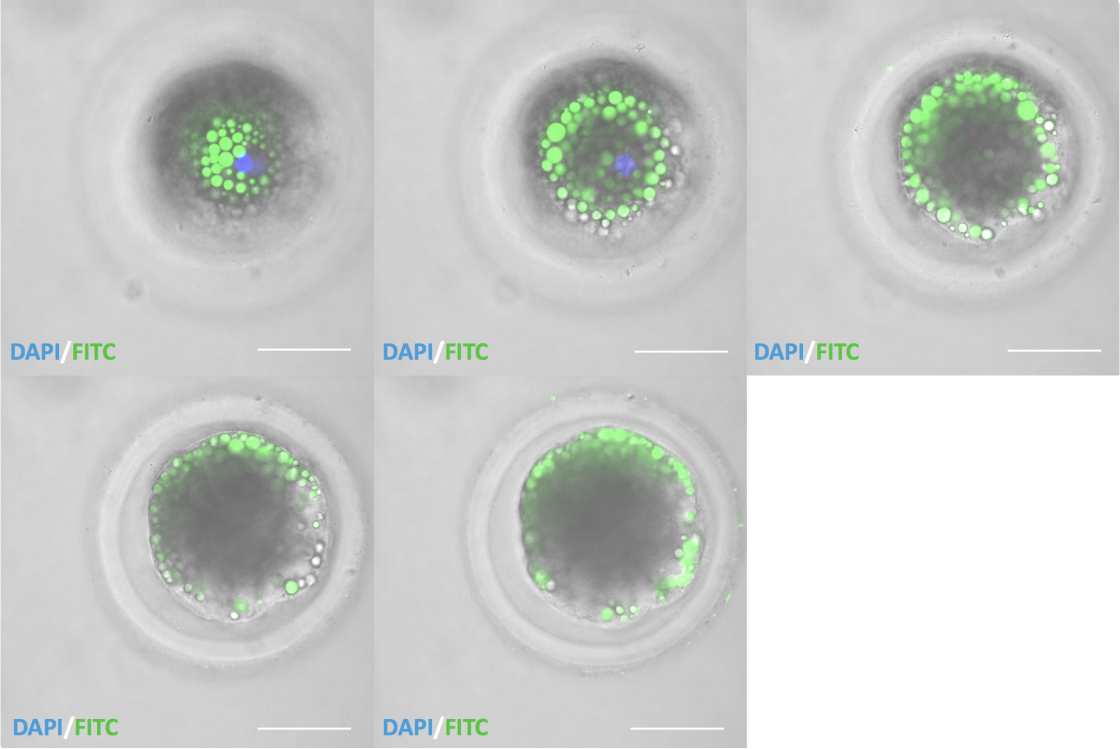


**Supplementary Figure 1.** Oocyte stacks taken with confocal microscope from one pole to the equatorial section. Oocytes were stained with Bodipy 493/503 (FITC) for lipid droplets and DAPI (blue) for chromatin visualization; scale bar represent 50μm, sections were taken every 10μm.


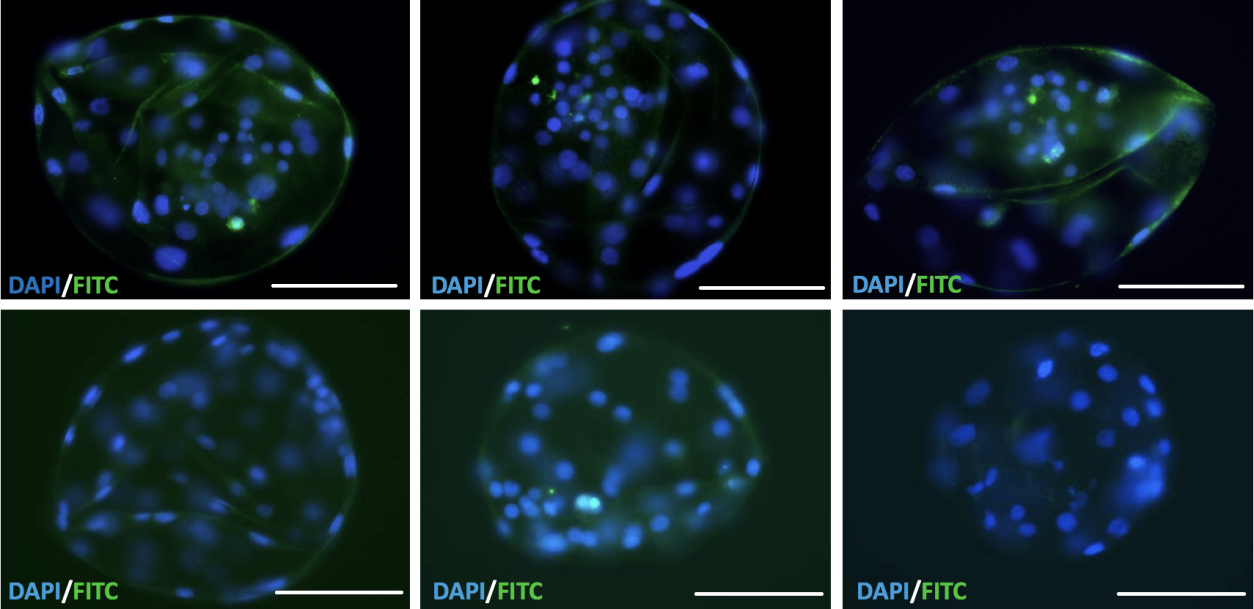


**Supplementary Figure 2.** Apoptosis analysis in parthenogenetic porcine blastocysts (7 days post activation) stained with DAPI (nucleus) and fluorescein-conjugated dUTP mix (apoptotic nucleus – FITC — green). Scale bars represent 100μm.


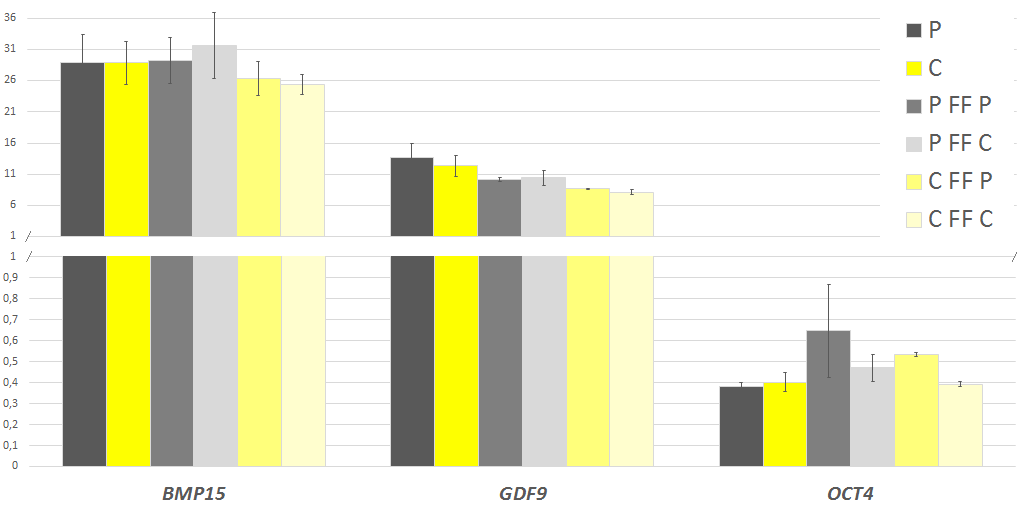


**Supplementary Figure 3.** mRNA expression level of genes related to developmental competence in oocytes before *in vitro* maturation and oocytes matured *in vitro*.. P – oocytes from prepubertal gilts; C – oocytes from cyclic gilts; P FF P – oocytes collected from prepubertal females matured with autologous, prepubertal follicular fluid; P FF C - oocytes collected from prepubertal females matured with follicular fluid from cyclic gilts; C FF P - oocytes collected from cyclic females matured with follicular fluid from prepubertal gilts; C FF C - oocytes collected from cyclic females matured with autologous, cyclic follicular fluid


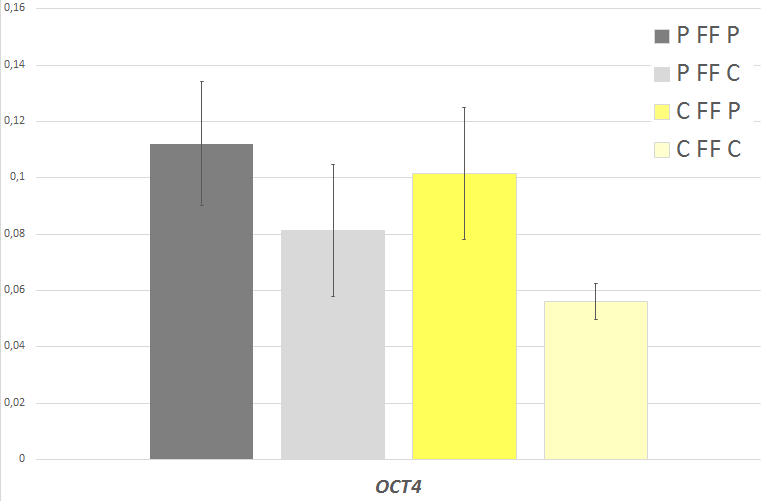


**Supplementary Figure 4.** mRNA expression level of OCT4 in parthenogenetic porcine blastocyst in all experimental groups. P FF P – blastocysts obtained from oocytes of prepubertal females matured with autologous, prepubertal follicular fluid; P FF C - blastocysts obtained from oocytes of prepubertal females matured with follicular fluid from cyclic gilts; C FF P - blastocysts obtained from oocytes of cyclic females matured with follicular fluid from prepubertal gilts; C FF C - blastocysts obtained from oocytes of cyclic females matured with autologous, cyclic follicular fluid
